# Elucidating macular structure–function correlations in glaucoma

**DOI:** 10.1038/s41598-022-13730-z

**Published:** 2022-06-23

**Authors:** Sara Giammaria, Glen P. Sharpe, Oksana Dyachok, Paul E. Rafuse, Lesya M. Shuba, Marcelo T. Nicolela, Jayme R. Vianna, Balwantray C. Chauhan

**Affiliations:** 1 Department of Ophthalmology and Visual Sciences, Dalhousie University and Nova Scotia Health Authority, 1276 South Park Street, Victoria Building, Room 2035, Halifax, NS B3H 2Y9 Canada; 2 DSCMT, University of Rome Tor Vergata, Rome, Italy

**Keywords:** Diseases, Eye diseases, Glaucoma

## Abstract

Correlation between structural data from optical coherence tomography and functional data from the visual field may be suboptimal because of poor mapping of OCT measurement locations to VF stimuli. We tested the hypothesis that stronger structure–function correlations in the macula can be achieved with fundus-tracking perimetery, by precisely mapping OCT measurements to VF sensitivity at the same location. The conventional 64 superpixel (3° × 3°) OCT grid was mapped to VF sensitivities averaged in 40 corresponding VF units with standard automated perimetry (conventional mapped approach, CMA) in 38 glaucoma patients and 10 healthy subjects. Similarly, a 144 superpixel (2° × 2°) OCT grid was mapped to each of the 68 locations with fundus-tracking perimetry (localized mapped approach, LMA). For each approach, the correlation between sensitivity at each VF unit and OCT superpixel was computed. Vector maps showing the maximum correlation between each VF unit and OCT pixel was generated. CMA yielded significantly higher structure–function correlations compared to LMA. Only 20% of the vectors with CMA and < 5% with LMA were within corresponding mapped OCT superpixels, while most were directed towards loci with structural damage. Measurement variability and patterns of structural damage more likely impact correlations compared to precise mapping of VF stimuli.

## Introduction

Optical coherence tomography (OCT) makes it possible to image and segment the multilayered structure of the macula. Because approximately 40% of retinal ganglion cells (RGCs) are located within the macula^1^, there has been a recent focus on macular OCT imaging to measure the reduction in the thickness of inner retinal layers caused by glaucoma^2–4^, in manner that approximates histological analysis^5^. Loss of RGCs also results in loss of visual field (VF) sensitivity estimated with standard automated perimetry.

However, studying the relationship between structural and functional loss in the macula is challenging, due mainly to three reasons that affect the agreement between VF sensitivity and the corresponding loss with OCT. First, the relative areas of comparison are different as the VF is conventionally measured with a size III stimulus, subtending an area of 0.43°^6^, while standard OCT sectors measure retinal thickness in larger areas, which vary in size according to the different devices. Second, there is low spatial correspondence between stimulated photoreceptors and corresponding RGCs in the central retina as the latter are anatomically displaced^1,7^, with up to 2° of transverse displacement in the case of the four central VF locations of the 10–2 test pattern of standard automated perimetry^7,8^. Third, there is considerable test–retest variability in both structural and functional measurements^9–16^, which can compound the measurement errors and impact the correlation.

While modern OCT devices have incorporated mechanisms to correct for eye movements to reduce the measurement error at a specific imaged location, this is not the case with standard automated perimetry. Although fixation error checks give an indication of fixation accuracy, there can be significant errors (theoretically subtending the extent of the blind spot) that result in the stimulus not being projected on the intended location in the retina, reducing the reliability of VF sensitivity estimates^17^. To overcome this issue, new fundus-tracking perimeters have recently become commercially available. They are designed to compensate for eye movements by tracking the retina during the examination^18^, and presenting the stimulus in the same retinal location, thereby, minimizing the component of variability due to fixation instability^19^.

In an attempt to establish the correspondence between structural and functional measurements, the conventional method of mapping OCT measurements to VF locations (hereafter termed, conventional mapped approach, CMA), relies on relating measurements averaged either within standard OCT sectors or blocks of pixels (termed, superpixels) to VF sensitivity in corresponding groups of VF locations of standard automated perimetry^20–22^. Additionally, models are used to account for the non-alignment of photoreceptors and RGCs in the central retina by displacing the mapped VF locations^7,8,23^. However, with standard automated perimetry, variability in the projection of the VF stimulus on the retina due to inaccurate fixation is a factor and may impair the veracity and the strength of the structure–function relationship assessed with this approach.

In this study we used fundus-tracking perimetry and techniques that precisely map OCT measurements in highly localized areas to VF sensitivity at the same location (hereafter termed, localized mapped approach, LMA). We wanted to test the hypothesis that in patients with open-angle glaucoma and healthy control subjects, a stronger structure–function relationship in the macula could be achieved with LMA compared to CMA.

## Results

### Sample description and segmentation accuracy

There were 38 glaucoma patients and 10 healthy subjects in the study. The demographic and baseline clinical characteristics of the study participants are reported in the Table. There was a wide range of central VF damage in the glaucoma patients with the 10–2 Mean Deviation (MD) ranging from − 21.99 to 0.33 dB.

Manual correction of automated segmentation was required in 52 (1.8%) of the total of 2928 B-scans of the posterior pole scan protocol used for the CMA and 232 (4.0%) of the total of 5808 B-scans of the high-density scan protocol used for the LMA.

### Structure–function spatial mapping

**Figure 1:**
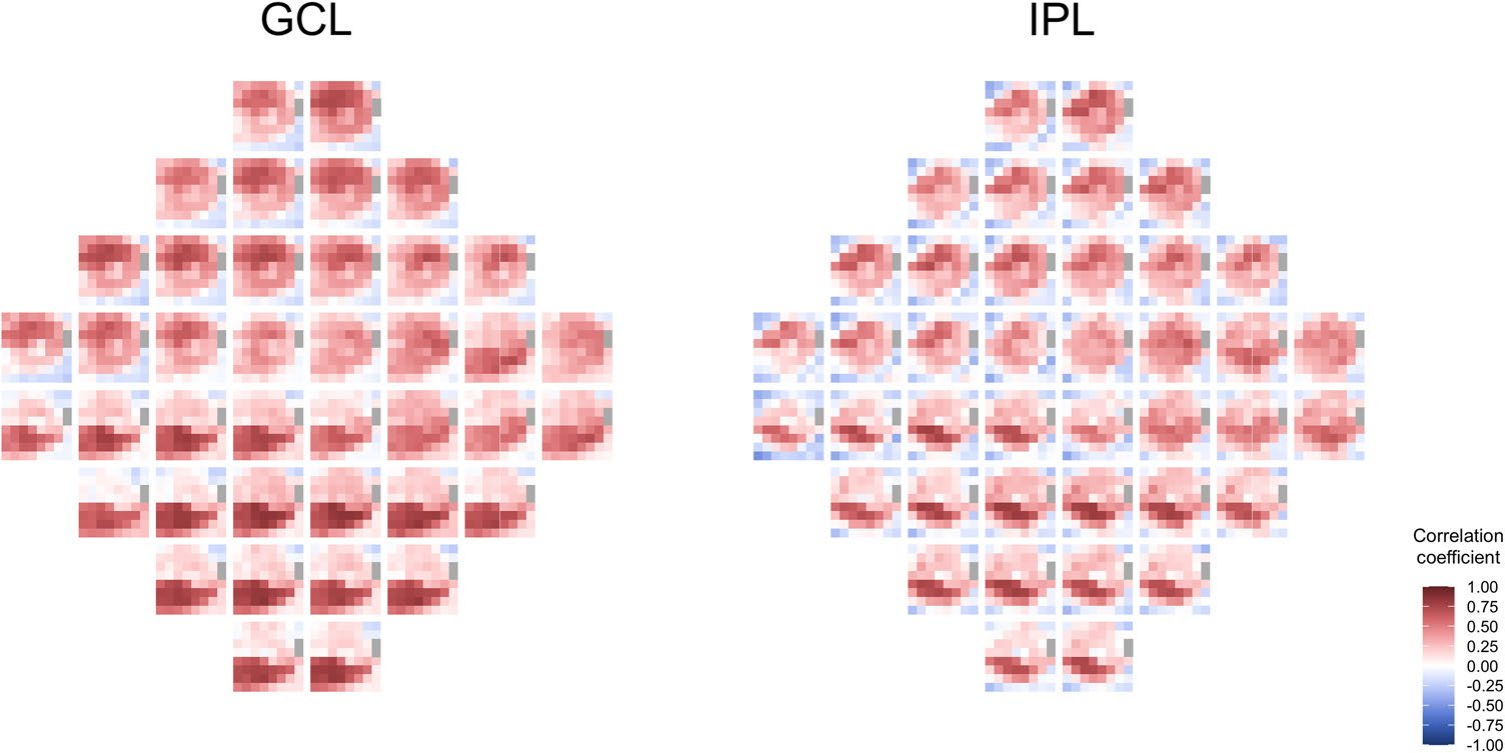
Heatmaps of Pearson’s correlation coefficients between visual field units and OCT superpixels for the conventional mapped approach (CMA). Heatmaps of Pearson’s correlation coefficients between visual field (VF) units and OCT superpixels in the ganglion cell layer (GCL) and inner plexiform layer (IPL) for the conventional mapped approach. Each of the 40 squares represents the heatmap of the correlations between a single VF unit with all the 64 OCT superpixels. The VF units were flipped to correspond to the appropriate hemimacula.

**Figure 2:**
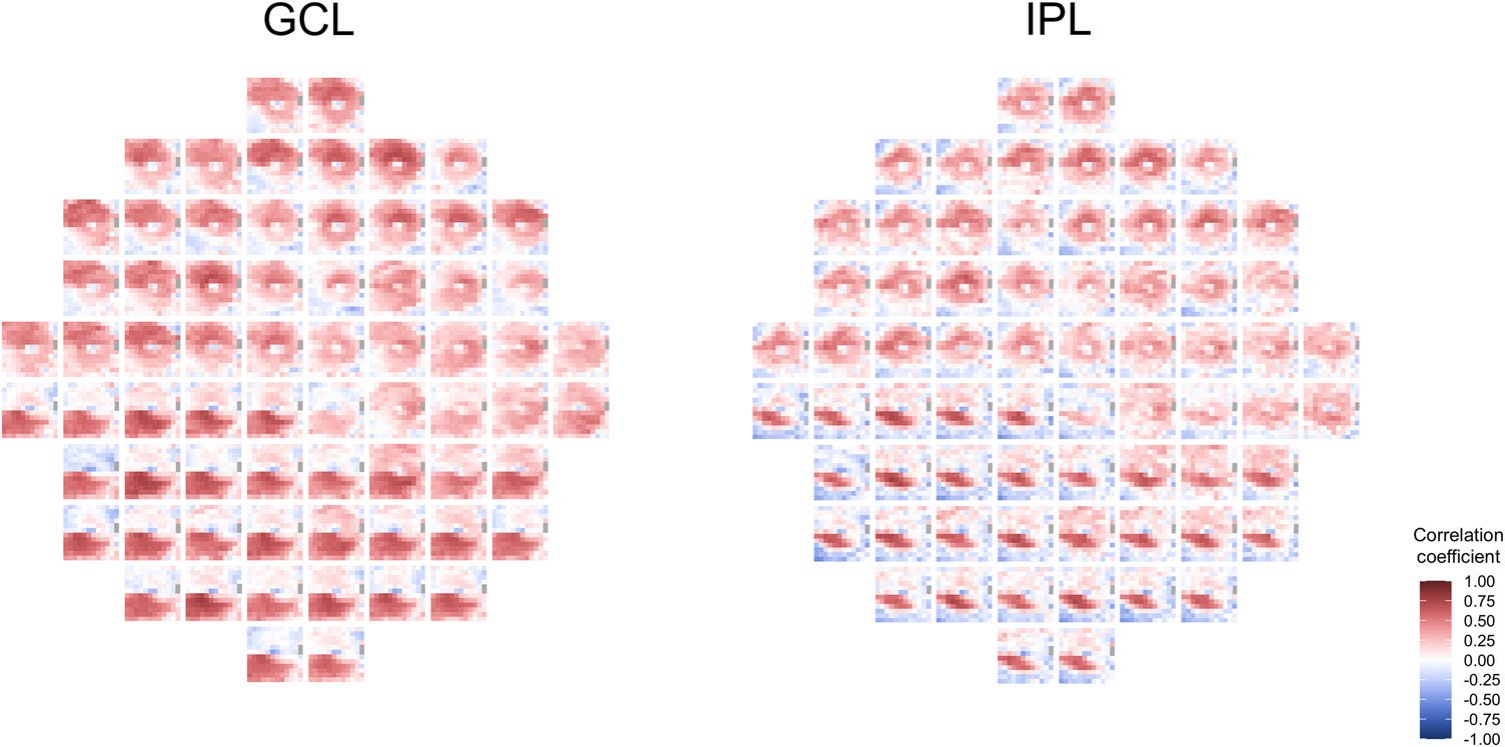
Heatmaps of Pearson’s correlation coefficients between visual field units and OCT superpixels for the localized mapped approach (LMA). Heatmaps of Pearson’s correlation coefficients between visual field (VF) units and OCT superpixels in the ganglion cell layer (GCL) and inner plexiform layer (IPL) for the localized mapped approach. Each of the 68 squares represents the heatmap of the correlations between a single VF unit with all the 144 OCT superpixels. The VF units were flipped to correspond to the appropriate hemimacula.

Figure 1 shows the heatmaps of the correlation coefficients between sensitivity in VF units (flipped to correspond to the appropriate hemimacula) and thickness measurements in OCT superpixels for the CMA. For both the GCL and IPL, positive correlations had a “bull’s eye” pattern and was particularly evident in the IPL where lower positive and negative correlations were most frequently located in the peripheral and foveal superpixels. The inferior VF units had a higher correlation with superpixels in the superior hemimacula and, conversely, the superior VF units with superpixels in the inferior hemimacula. Furthermore, the correlation between the superior VF units and the inferior superpixels was higher than that between the inferior VF units and superior superpixels (Fig. 1). Correlations for the LMA also had a bull’s eye pattern (Fig. 2) and the spatial distribution of the correlations was similar to that noted for the CMA.

**Figure 3:**
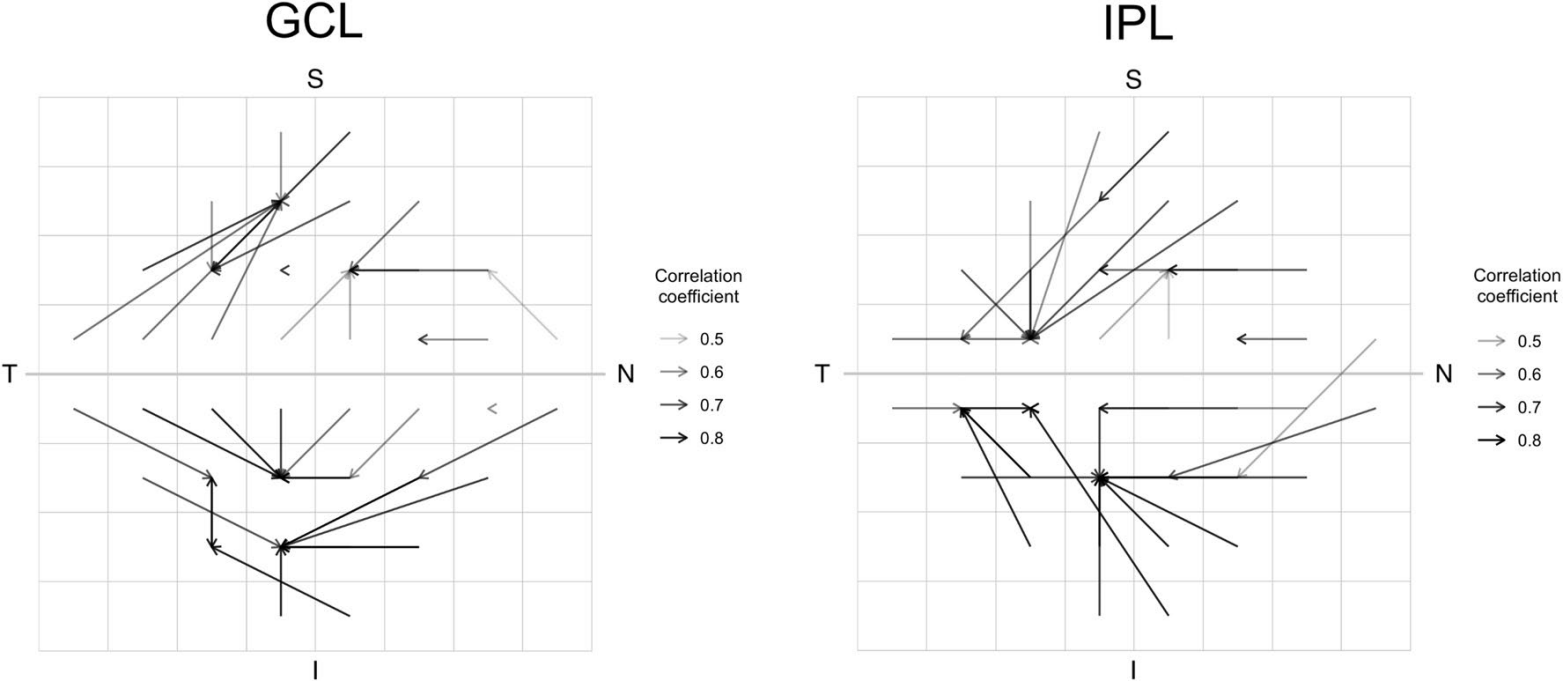
Vector maps of the correlation coefficients obtained with the conventional mapped approach (CMA). Vector maps of the correlation coefficients in ganglion cell layer (GCL) and inner plexiform layer (IPL) obtained with the conventional mapped approach. Vectors connect each of the 40 visual field units with the OCT superpixel with the maximum correlation coefficient. The gray scale of the vectors represents the strength of the correlations. The bold gridline indicates the horizontal midline. S = Superior; N = Nasal; I = Inferior; T = Temporal.

**Figure 4:**
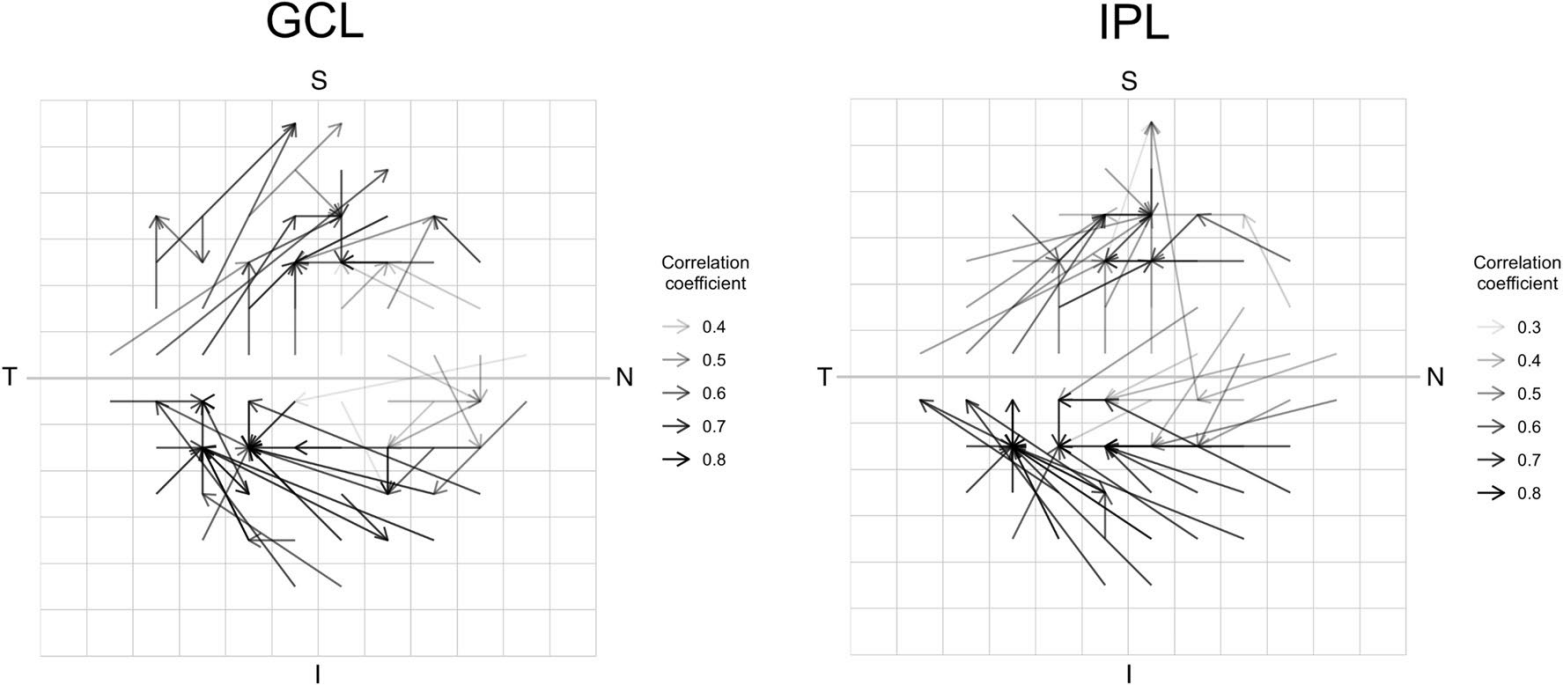
Vector maps of the correlation coefficients obtained with the conventional mapped approach (LMA). Vector maps of the correlation coefficients in ganglion cell layer (GCL) and inner plexiform layer (IPL) obtained with the localized mapped approach. Vectors connect each of the 68 visual field units with the OCT superpixel with the maximum correlation coefficient. The gray scale of the vectors represents the strength of the correlations. The bold gridline indicates the horizontal midline. S = Superior; N = Nasal; I = Inferior; T = Temporal.

The vector maps showing the maximum correlation between measurements in each VF unit and superpixels for the CMA and LMA are shown in Figs. 3 and 4, respectively. Of the 40 vectors (each corresponding to one VF unit) for the CMA, only 8 (20%) were directed to the corresponding mapped OCT superpixel in either the GCL or IPL (Fig. 3 and Supplementary Fig. 1). Of 68 vectors for the LMA, only 3 (4%) in GCL and 2 (3%) in IPL (Fig. 4 and Supplementary Fig. 2) were directed to the corresponding mapped OCT superpixel. For the CMA, vectors with length > 0° had a median length of 6° (range: 3°–12.7°) in the GCL and 7.6° (range: 3°–10.8°) in the IPL (Supplementary Fig. 1). For the LMA, vectors with distance > 0° had a median length of 4° (range: 2–12.8°) in the GCL and 4.5° (range: 2–12.2°) in the IPL (Supplementary Fig. 2).

The sample mean sensitivity at each VF unit, and GCL and IPL thickness at each superpixel for both CMA and LMA are shown in Supplementary Fig. 3, While the vectors generally did not cross the horizontal midline (Figs. 3 and 4), when they did, the correlations were relatively weak. For both CMA and LMA, multiple vectors were directed to single superpixels with relatively reduced mean GCL and IPL thickness (Supplementary Fig. 3), indicating that that these loci influenced the correlations, irrespective of the location of the VF unit.

### Structure–function correlations

**Figure 5:**
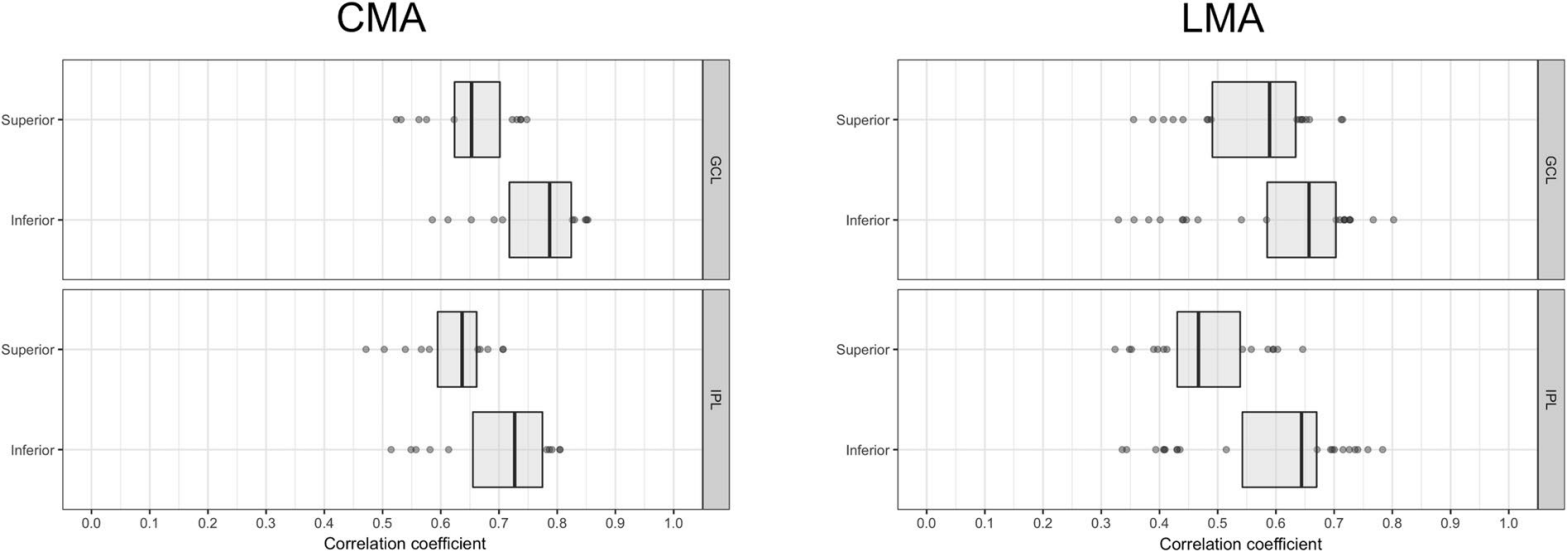
Distribution of the Pearson’s correlation coefficients in superior and inferior hemimaculas. Distribution of the Pearson’s correlation coefficients in superior and inferior hemimaculas in the ganglion cell layer (GCL) and inner plexiform layer (IPL) obtained with the conventional (CMA) and the localized (LMA) mapped approaches. Boxes represent the first and third quartiles and the vertical lines across the boxes indicate the medians.

The distribution of the maximum correlation coefficient obtained in each superpixel (for both the GCL and IPL) in the superior and inferior hemimaculas is shown in Fig. 5. The median value in the inferior hemimacula was higher than that of the superior hemimacula in both layers with the CMA: 0.65 compared with 0.78 for the GCL, and 0.63 compared with 0.72 for the IPL, respectively, in superior and inferior hemimacula (Fig. 5). Higher correlation coefficients in the inferior hemimacula were also obtained with the LMA: 0.59 compared with 0.65 in the GCL, and 0.46 compared with 0.64 in IPL, respectively (Fig. 5).

**Figure 6:**
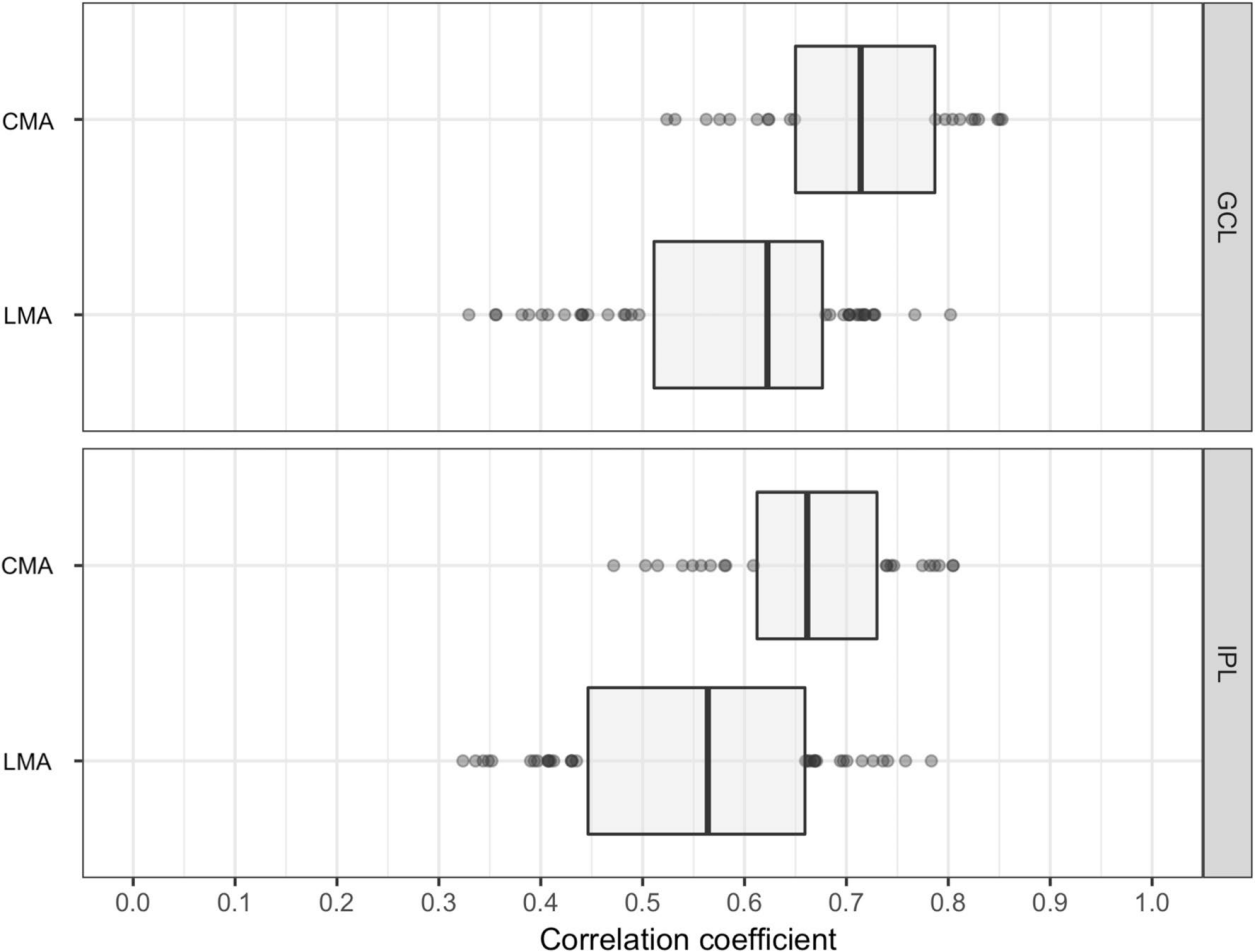
Distribution of the Pearson’s correlation coefficients obtained with the conventional (CMA) and the localized (LMA) mapped approaches. Distribution of the Pearson’s correlation coefficients in the ganglion cell layer (GCL) and inner plexiform layer (IPL) obtained with the conventional (CMA) and the localized (LMA) mapped approaches. Boxes represent the first and third quartiles and the vertical lines across the boxes indicate the medians.

For both the GCL and IPL, the CMA yielded higher correlation coefficients compared to the LMA [median(range): 0.71 (0.52–0.85) compared with 0.62 (0.32–0.80) for the GCL (*p* < 0.001) and 0.66 (0.47–0.80) compared with 0.56 (0.32–0.78) for the IPL (*p* < 0.001), respectively; Fig. 6].

## Discussion

The study of structure and function correlations in the macula by combining OCT imaging and VF assessment is thought to be important for understanding the nature of glaucomatous damage and for strengthening the basis for diagnostic decisions. Additionally, data from macular testing can provide supplementary evidence to optic nerve head and retinal nerve fibre layer examination, particularly when a differential diagnosis has to be made, for example, in cases of high myopia^24–26^. However, evidence to date has not provided a clear and unambiguous foundation for understanding how structural and functional damage are related to each other. In fact, methodologies of CMAs that examine this relationship have yielded at best moderate correlations with maximum correlation coefficients ranging from 0.41 to 0.77^20–22,27–29^. The present study was carried out to map the macular structure–function relationship by comparing conventional techniques to one employing fundus-tracking perimetry to accurately map each VF test location to corresponding locations in the retina to correlate VF sensitivity to GCL and IPL thickness values. However, our hypothesis that the LMA would yield higher correlations between VF sensitivity and OCT thickness values was not substantiated.

Our hypothesis was based on the assumption of accurate correspondence between the retinal location where the VF stimulus was projected and the retinal location where OCT measurements were made. We centred the GCL and IPL thickness values on each VF stimulus to ensure that measurements were localized. Based on a similar premise, but different methodologies, a comparable^30^ or even better^31^ structure–function relationship in the macula was reported with fundus-tracking perimetry. Contrary to these studies, our results showed that the correlation coefficients obtained with the LMA were significantly lower compared to the CMA.

Many previous reports on the structure–function relationship in the macula only explored the correlations between standard OCT sectors or superpixels and corresponding VF locations, constraining the correlations only to mapped locations to maintain the concordance between the anatomical distribution of RGCs and VF locations^20–22,27–29^. In contrast, we explored correlations over the entire macula instead of exclusively between VF units and corresponding OCT superpixels. We found that most of the vectors that linked each VF unit to the OCT superpixel with the maximum correlation were longer than expected and directed towards retinal locations further from the theoretical mapped location. These findings were observed for both for the CMA (80% each for the GCL and IPL; Fig. 3 and Supplementary Fig. 1) and LMA (96% and 97% for GCL and IPL respectively; Fig. 4 and Supplementary Fig. 2).

Outside the central 8° of the macula, corresponding to approximately 2 mm from the fovea, the thickness of GCL and IPL decreases rapidly because the RGC somas are arranged in a single layer^1^. Due to the normally lower GCL and IPL thickness in these areas, glaucoma is unlikely to result in further significant reductions in thickness. Hence, the GCL and IPL thickness in unaffected areas with corresponding normal VF sensitivities may be similar to those in areas damaged by glaucoma that have corresponding reduced VF sensitivity^32^. Consequently, outside the 8° of the macula, very weak or even negative correlations were observed (Figs. 1 and 2), confirming previous findings^28,32^. In contrast, within the central 8°, where the RGCs are stacked within multiple layers and where the GCL and IPL thickness is greater^1^, glaucomatous damage produces progressive measurable thinning over a larger range of values. This progressive thinning, together with a corresponding reduction in VF sensitivity, yields a statistically higher correlation between structure and function. Lee and colleagues^28^, who used a sector based analysis, also found that maximum correlations tend to be concentrated in areas with a wider range of measurements and where glaucomatous damage can be more readily detected.

The CMA used 3° × 3° OCT superpixels, while the LMA used 2° × 2° superpixels. The different resolution of the two approaches may further explain the unexpected higher correlation observed with the CMA. In the latter, a varying number of VF locations were used to derive VF units within which VF sensitivity was averaged. Furthermore, OCT superpixels were larger compared to the LMA where VF units were single VF locations and OCT superpixels were smaller. Averaging VF sensitivities, and GCL and IPL thickness over larger areas may have resulted in higher signal-to-noise ratio and consequently higher correlation coefficients with the CMA compared to the LMA. In order to test the hypothesis that higher correlations resulted because of averaging over a larger area, we performed a LMA-like analysis with standard automated perimetry and a CMA-like analysis with fundus-tracking perimetry and showed that for both types of perimetry, averaging over a larger area resulted in higher correlations (Supplementary Figs. 4 and 5, respectively).

The differences in the size of OCT superpixels and VF units between the two approaches could have affected the degree of error in vector direction and length. As result, we observed more vectors with spurious directions with the LMA compared to the CMA.

Another factor that may explain the lower correlations obtained with LMA could be the greater experience subjects had with standard automated perimetry compared to fundus-tracking perimetry. Subjects were recruited from a prospective study in which they are regularly tested with standard automated perimetry. While unreliable tests with fundus-tracking perimetry were repeated when unreliable, we cannot rule out the possibility that learning effects or inadequate experience may have influenced the correlations obtained with the LMA. Differences in thresholding strategies between the two perimeters may have impacted the observed correlations. To test this hypothesis, we compared the CMA with the CMA-like approach with fundus-tracking perimetry and the LMA with the LMA-like approach (used in Supplementary Figs. 4 and 5). In both cases, we showed that standard automated perimetry always yielded higher correlation coefficients (Supplementary Figs. 6 and 7), irrespective of CMA or LMA.

For both the CMA and LMA, we noted more vectors directed temporally in the inferior retina (corresponding to the superior VF) compared to the superior retina (Figs. 3 and 4). The infero-temporal sector is considered to be susceptible and where damage is thought to occur early in the disease^2,33,34^. These observations support the notion that the pattern of glaucomatous damage may drive the magnitude of maximum correlations and vector direction when studying the structure–function relationship. According to topographic models, nerve fibre bundles from the superior hemiretina take a more peripheral trajectory compared to those from the inferior hemiretina which course closer to the fovea^35,36^. Therefore, damage to fibres in the superior retina could lead to a loss of RGCs further from the fovea and correspond to more peripheral inferior VF defects that may fall outside the central 10°. Conversely, damage to fibres in the inferior retina would correspond to superior VF defects closer to fixation inside the central 10°, and therefore could have led to a higher structure–function correlation inferiorly (Fig. 5), as also reported by others^22,27–30,37,38^.

In most of the retina, photoreceptors are vertically aligned with the RGCs, however in the central 8–9°^1^, because of the high concentration of cones and the foveal pit, corresponding RGCs are eccentrically displaced. For this reason, displacement models were conceived for more accurately mapping the projection of VF locations to RGCs corresponding to the stimulated photoreceptors^7,8^. Theoretically, the maximum correlation between either GCL or IPL thicknesses and VF sensitivity should lie at the location identified by the displacement models. Additionally, studies using displacement models in structure–function analysis should yield higher correlations compared to those that do not use them. However, evidence to date indicates that application of displacement models fails to unequivocally demonstrate a significant increase in correlation^28,38,39^. Indeed, our findings showed that the average length of the correlation vectors was considerably greater than the highest displacement proposed by the models, which range from 1° to 3°^7,8,40^.

Our study is limited by the relatively small sample size, mainly due to the requirement of additional inclusion criteria that placed subjects into specific mean GCL thickness and 10–2 MD tertiles. We decided to add these criteria to ensure an adequate sample size to represent the range of glaucomatous damage. However, we acknowledge that inclusion of patients with very early glaucoma where measurements are not substantially different to age-matched control values, could have led to a weaker structure–function relationship^42^. Likewise, in advanced glaucoma, correlations may be inaccurate because the lower end of the measurement range of the perimeters and OCT devices may be reached at different stages of the disease^20,42^. Hence for a given patient, the veracity of VF and OCT measurements could be variable. Studying the impact of fundus tracking on the structure–function correlations could have been performed by comparing results with and without the tracking activated, as performed previously^43^, however, the primary goal of this study was study the conventional mapping with the HFA.

In summary, contrary to our hypothesis, the LMA, using fundus-tracking perimetry to accurately map VF locations to corresponding GCL and IPL thickness values, did not improve the structure–function correlations in the macula. The poor correlation of the CMA is less likely due to inaccurate mapping of VF locations to the retina, but more likely due to factors such as variability in measurements, which affect both forms of perimetry and OCT and specific patterns of localized damage that drive vector direction and strength of maximum correlations. Clinical judgment that subjectively relates structural and functional losses in the macula in relatively large areas of analysis remain valuable. For example, decreased inferior macular GCL and IPL thickness is useful for corroborating superior macular visual field loss. However, the findings of this study question the utility of analyses involving macular structure–function correlations, performed in an objective and quantitative manner within smaller areas, for aiding clinical decision making in glaucoma.

## Methods

The participants were glaucoma patients enrolled from ongoing prospective studies on detecting the earliest progression of open-angle glaucoma with imaging and perimetry, and quantifying age-related changes in parallel cohorts of healthy control subjects. The study received ethics approval from the Nova Scotia Health Authority Research Ethics committee and in accordance with the tenets of Declaration of Helsinki all participants gave informed consent.

### Participants

Glaucoma patients were recruited consecutively from glaucoma clinics at the Eye Care Centre of the Nova Scotia Health Authority, while healthy control subjects were recruited from spouses or partners of patients and the non-hospital-based community through advertisements in the local media. Examinations from November 2020 to February 2021 were included in the analyses.

Inclusion criteria for glaucoma patients were: (1) visual acuity ≥ 6/12; (2) clinical diagnosis of open-angle glaucoma; (3) optic nerve changes and VF damage compatible with glaucoma and (4) abnormal Glaucoma Hemifield Test. Inclusion criteria for healthy subjects were: (1) visual acuity ≥ 6/12; (2) normal eye examination; (3) intraocular pressure ≤ 21 mm Hg and (4) normal VF with the Glaucoma Hemifield Test within normal limits.

Glaucoma patients and healthy subjects were excluded if any of the following were present: (1) chronic ocular disease (except glaucoma in patients); (2) systemic disease or treatment capable of affecting the VF; or (3) refractive error exceeding 6 diopters equivalent sphere or 3 dioptres of astigmatism.

When both eyes were eligible, one eye per subject was randomly included in the study.

### Methodology for the CMA

The VF was tested with the 10–2 pattern of Humphrey Field Analyzer (HFA, Carl Zeiss Meditec, Dublin, CA) which contains 68 test locations 2° apart, both horizontally and vertically, within the central 10° of the VF. The appropriate near correction was used and fixation monitored with the Hejil-Krakau method^44^. Tests deemed unreliable as noted by the perimetrist and reliability criteria (false positive or false negative rates > 15%, or fixation losses > 33%) were repeated.

OCT examinations were performed with the Spectralis OCT2 (Heidelberg Engineering, Heidelberg, Germany). Images were acquired within the central 30° × 25° (Glaucoma Module Premium Edition software, GMPE, Heidelberg Engineering, v. 6.16) with 61 horizontal B-scans (each containing 768 A-scans) averaged 9 times. Automatic segmentation of the individual retinal layers was carried out to quantify ganglion cell layer (GCL) and inner plexiform layer (IPL) thickness, using the device software. Only scans with high signal strength (> 25 dB) were included in the analysis. All images were checked for segmentation errors and manually corrected by a trained operator when necessary.

### Methodology for the LMA

From the whole population of glaucoma patients and healthy subjects, we selected a subset of subjects with a wide range of both structural and functional damage to be additionally tested with the LMA. To identify this subset, we first computed tertiles of the distribution of the mean GCL thickness and 10–2 MD in all patients. The mean GCL thickness and MD ranges for the first, second and third tertile groups were ≤ 23 µm and ≤ − 10 dB; 24 to 27 µm and − 10 dB to − 4 dB; and ≥ 28 µm and ≥ − 4 dB, respectively. We then consecutively recruited an approximately equal number of glaucoma patients within each tertile group. We also selected a subset of healthy subjects to be tested with the LMA.

This subset of subjects was tested with the 10–2 pattern of the Compass fundus-tracking perimeter (CMP, CenterVue, Padova, Italy), which is identical to the HFA 10–2 pattern. The CMP is equipped with an autofocus feature so that a refractive correction is not required. Tests considered unreliable (false positive > 18%, false negative > 25% and blind spot response > 25%) were repeated. High density OCT scans within the central 30° × 25° with 121 B-scans (each containing 1536 A-scans), averaged 9 times were obtained. Automatic segmentation was carried out to quantify GCL and IPL thickness. Only scans with high signal strength (> 25 dB) were included in the analysis and all images were checked for segmentation errors and manually corrected by the same trained operator when necessary.

### Structure–function investigation

**Figure 7:**
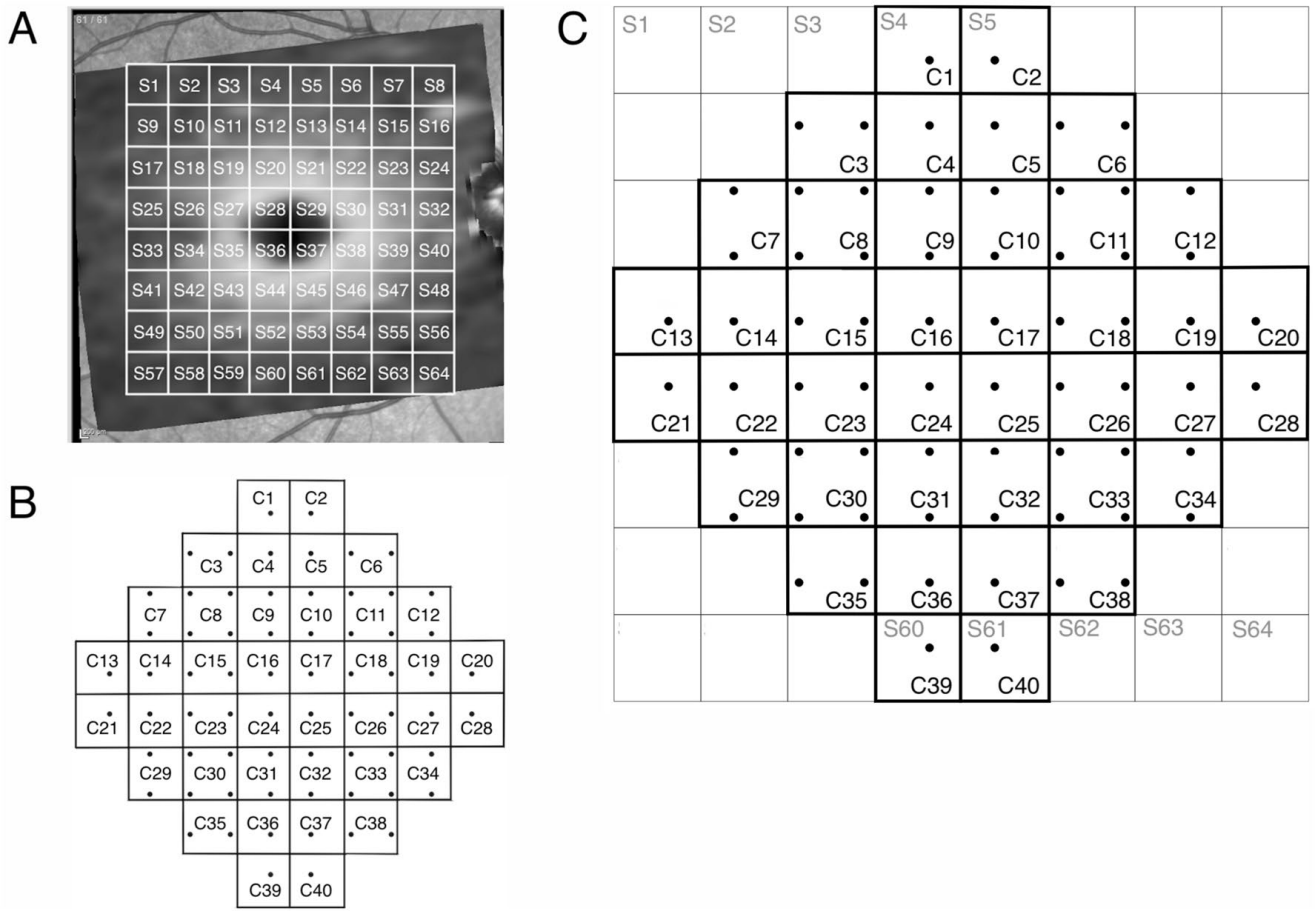
Methodology for the conventional mapped approach (CMA). Methodology for the conventional mapped approach comprising 64 OCT superpixels (S1–S64) arranged in an 8 × 8 superpixel grid (**A**) and 40 visual field (VF) units (C1–C40) (**B**) matching the OCT grid (**C**). The OCT grid is horizontally oriented, and the VF is flipped along the horizontal midline to correct for orientation.

For the CMA, the posterior pole analysis grid of the GMPE software was used. The output provides retinal layer thickness values, in a grid parallel to the fovea to Bruch’s membrane opening centre (FoBMO) axis, comprising a grid of 8 × 8 superpixels (64 in total, S1-S64, Fig. 7), with each superpixel corresponding to a 3° × 3° area. For each subject, the OCT superpixel grid was rotated according to the FoBMO angle such that the grid was horizontal in each eye (Fig. 7A). The GCL and IPL layer thickness values were averaged within each superpixel. The VF locations of the 10–2 pattern of the HFA were flipped along the horizontal midline to correspond to the OCT superpixel grid. There were 40 VF units (C1 to C40, Fig. 7B and C), containing between 1 and 4 VF locations, within which VF sensitivity was averaged.

**Figure 8:**
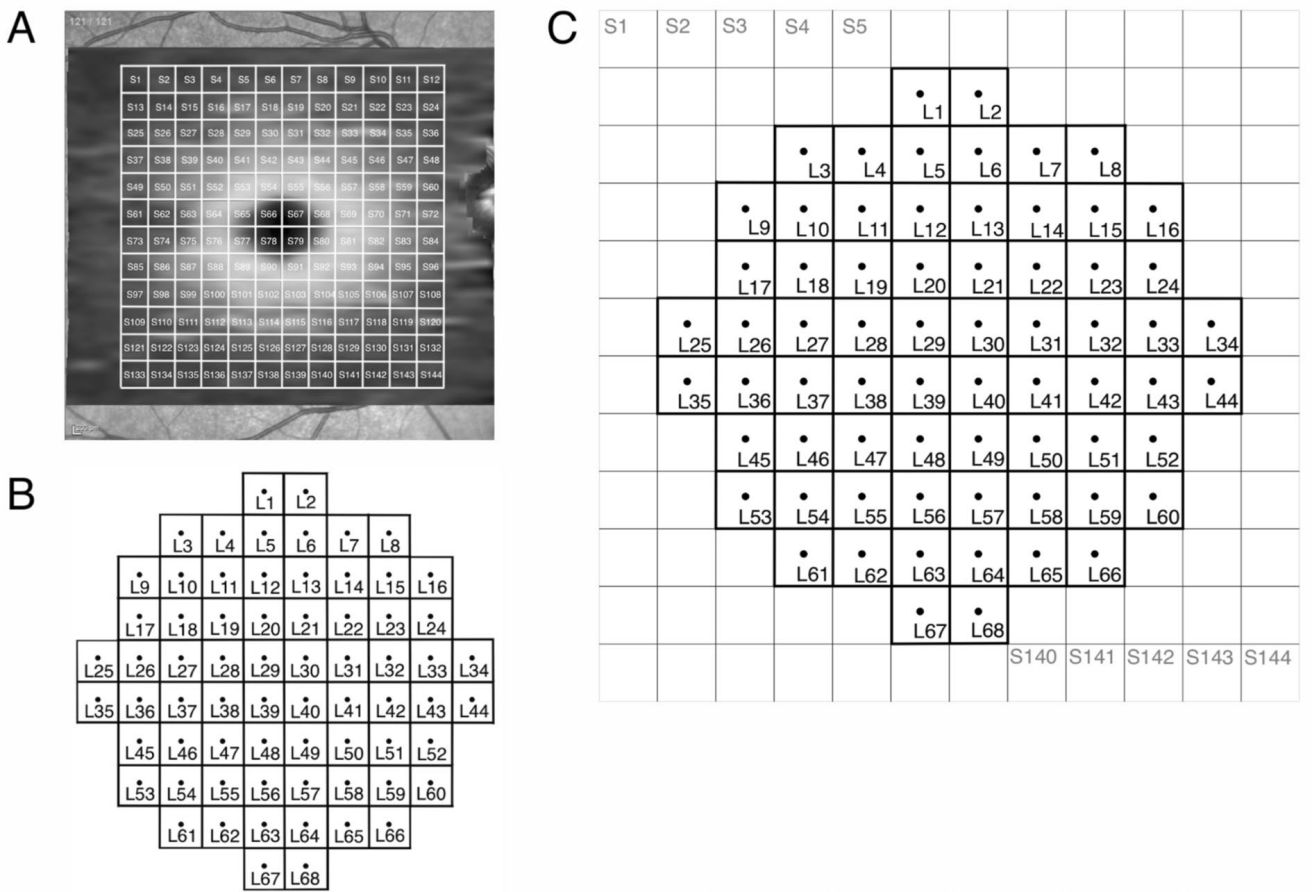
Methodology for the localized mapped approach (LMA). Methodology for the localized mapped approach comprising144 OCT superpixels (S1-S144) arranged in a 12 × 12 superpixel grid (**A**) and 68 visual field (VF) units (L1–L68) (**B**) matching the OCT grid (**C**). The VF is flipped along the horizontal midline to correct for orientation.

For the LMA, the infrared fundus image to which VF locations of the CMP were registered, was imported to the device software (SP-X1701 Spectralis Viewing Module v. 6.9.5.702, Heidelberg Engineering). The software precisely registered the infrared images from the CMP and the Spectralis (Supplementary Fig. 8) according to a transformation previously described^45^. In all registered images, rotation effects with respect to the horizontal axis were corrected accordingly. We divided the imaged area into a grid of 12 × 12 superpixels (144 in total, S1-S144, Fig. 8), with each superpixel subtending 2° × 2° (Fig. 8A). A 2° × 2° superpixel was chosen as the minimum size as the margin of error for subjects with unstable fixation during fundus-tracking perimetry is thought to be around 1.5°^46^. The GCL and IPL layer thickness values were averaged within each superpixel. Like with the CMA, the VF locations were flipped along the horizontal midline. In the LMA, each OCT superpixel was centered on each corresponding VF unit (each containing a single VF location, L1 to L68, Fig. 8B and C).

For both CMA and LMA, we excluded the superpixels in the corresponding to the optic nerve head to avoid spurious correlations due to missing values for GCL and IPL thickness. All data were converted to right eye format.

### Statistical analysis

We converted VF sensitivities from a logarithmic to linear scale according to the formula: *dB* = *10* × *log*_*10*_*(1/L)*^47^.

To map the structure–function relationship for the CMA, we computed the Pearson correlation coefficients of VF sensitivity obtained from all participants in each VF unit, with both GCL and IPL thickness values in each of the 64 OCT superpixels [for example, all the sensitivities obtained in VF unit 1 (C1) were correlated with all GCL and IPL thickness obtained in superpixel 1 (S1) through superpixel 64 (S64), Fig. 7C]. The analysis was then repeated for all remaining VF units. Similarly, for the LMA, the correlation coefficients of the sensitivity in one VF unit with both GCL and IPL thickness values in each of the 144 OCT superpixels of the customized posterior pole grid were computed [for example, the sensitivity in VF unit 1 (L1) was correlated with GCL and IPL thickness in superpixel 1 (S1) through superpixel 144 (S144), Fig. 8C]. The analysis was then repeated for all remaining VF units.

The correlation coefficients were expressed as heatmaps and used in the subsequent analyses. For each VF unit with each approach, we identified the coordinates of the OCT superpixel within which the GCL and IPL thickness was maximally correlated with VF sensitivity to derive a vector map. For each VF unit, the vector map illustrated the proximity of the retinal location where the structural measurements best corresponded with VF sensitivity. Correlation coefficients were compared with Wilcoxon signed-rank test after Fisher z-transformation. Statistical significance was defined at *p* < 0.05.

For statistical analysis, we used the open-source software R (V.3.6.0, R Core Team 2019; R: A language and environment for statistical computing. R Foundation for Statistical Computing, Vienna, Austria. URL https://www.R-project.org/) and R Studio (RStudio Team 2020; RStudio: Integrated Development for R. RStudio, PBC, Boston, MA. URL http://www.rstudio.com/).

## Supplementary Information

Supplementary Information 1.

Supplementary Information 2.

Supplementary Information 3.

Supplementary Information 4.

Supplementary Information 5.

Supplementary Information 6.

Supplementary Information 7.

Supplementary Information 8.
