# Supplementary figures and images for: Elucidating macular structure–function correlations in glaucoma
Source: Sci Rep. 2022 Jun 23;12:10621. doi: 10.1038/s41598-022-13730-z (PMC9226060; doi:10.1038/s41598-022-13730-z)

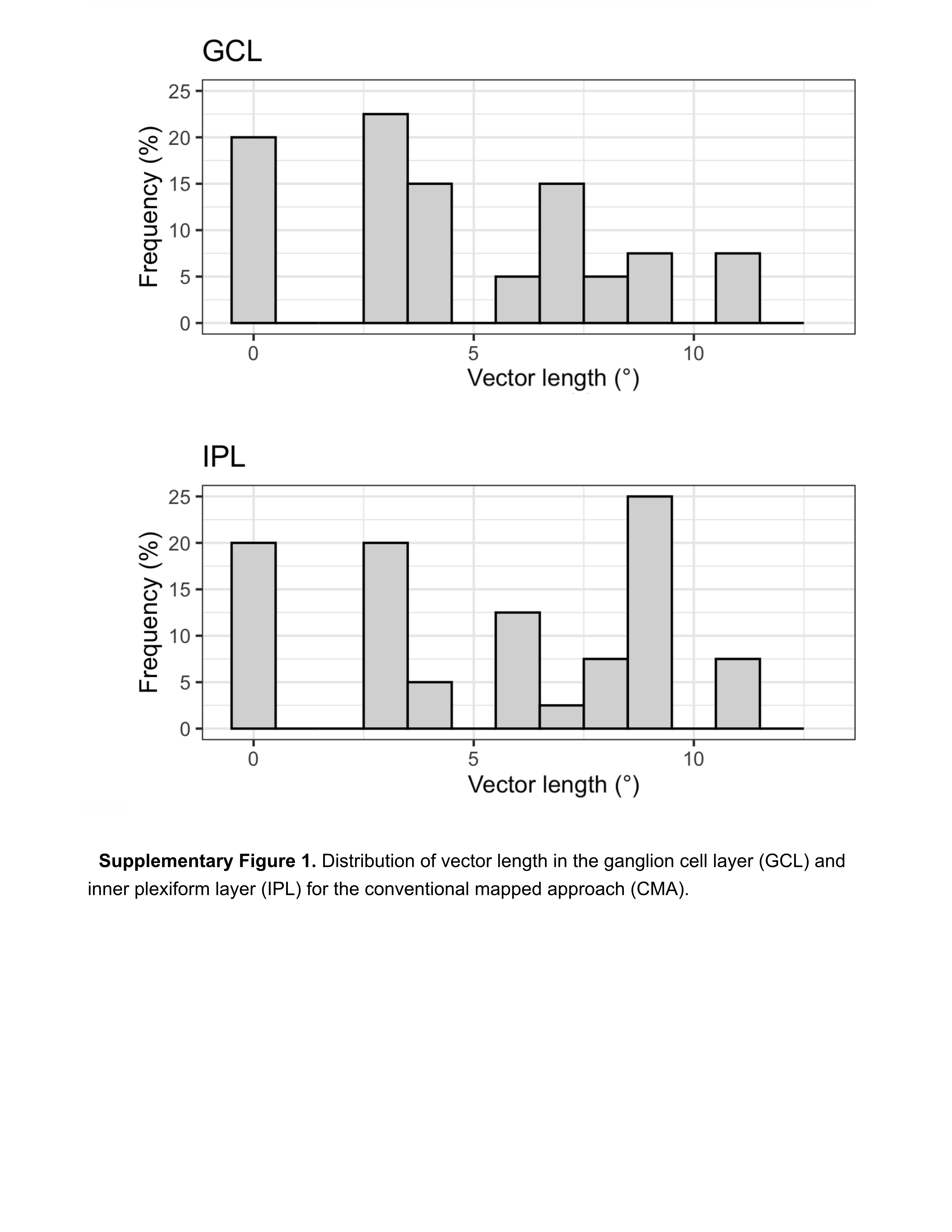

Supplement: Supplementary file 1 — Supplementary Information 1. [file 41598_2022_13730_MOESM1_ESM.tiff]

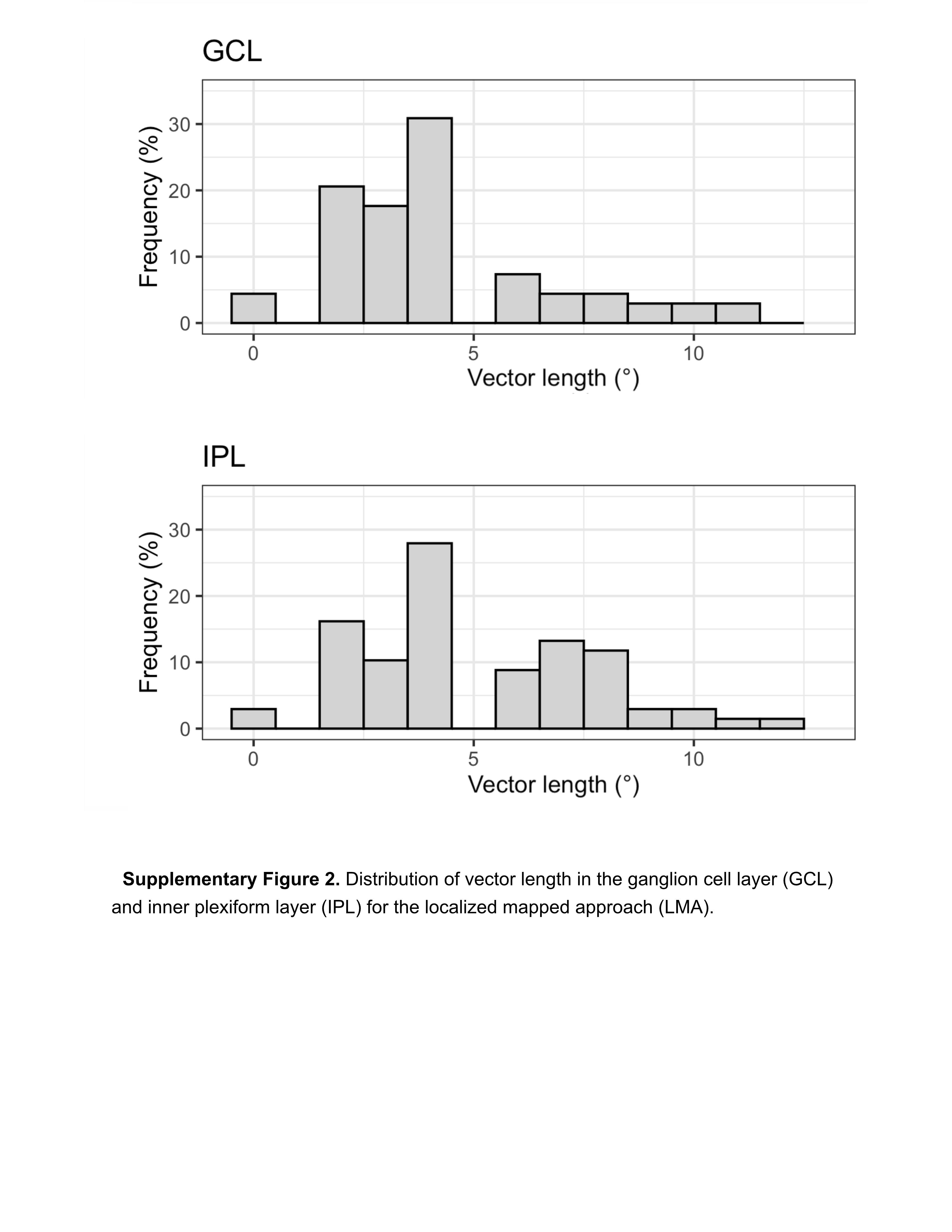

Supplement: Supplementary file 2 — Supplementary Information 2. [file 41598_2022_13730_MOESM2_ESM.tiff]

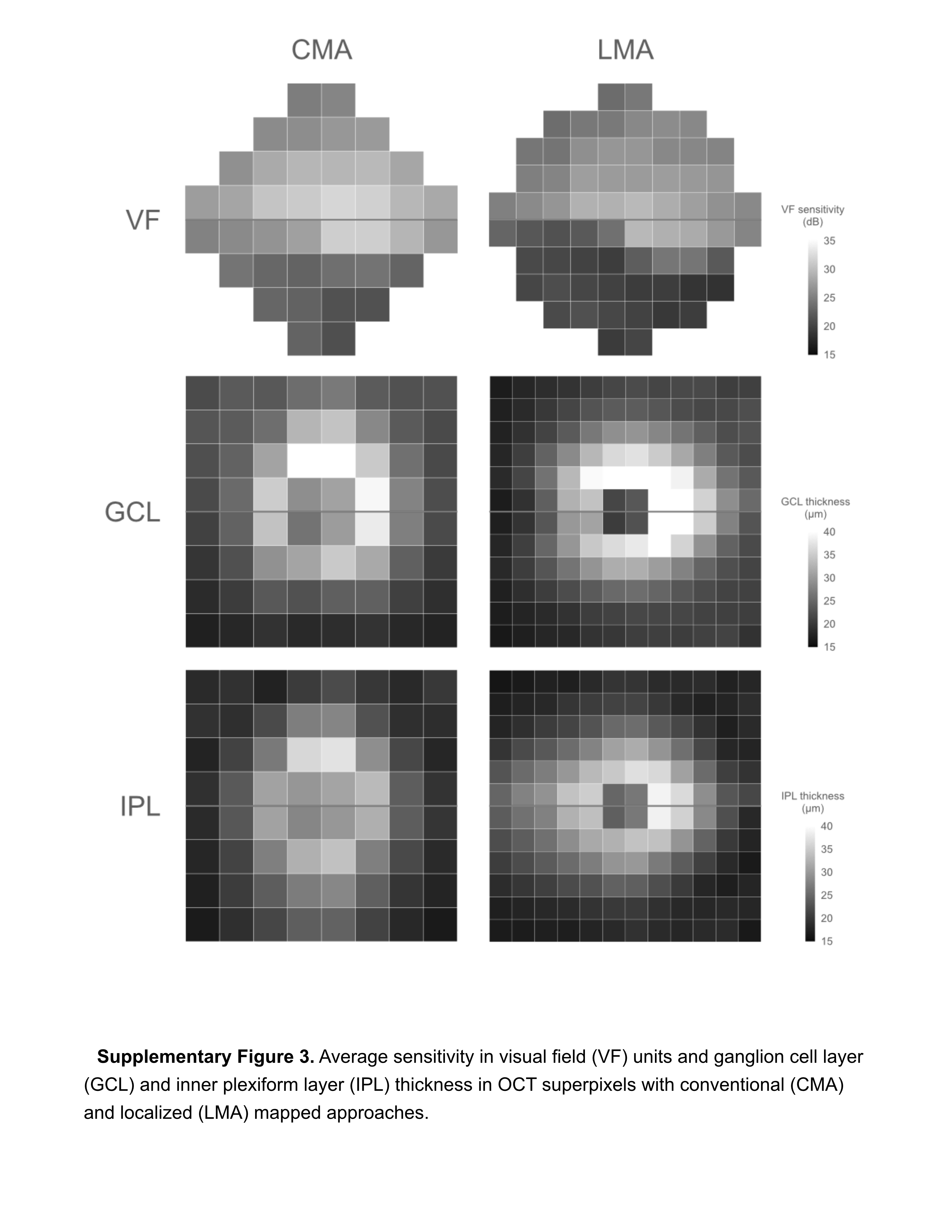

Supplement: Supplementary file 3 — Supplementary Information 3. [file 41598_2022_13730_MOESM3_ESM.tiff]

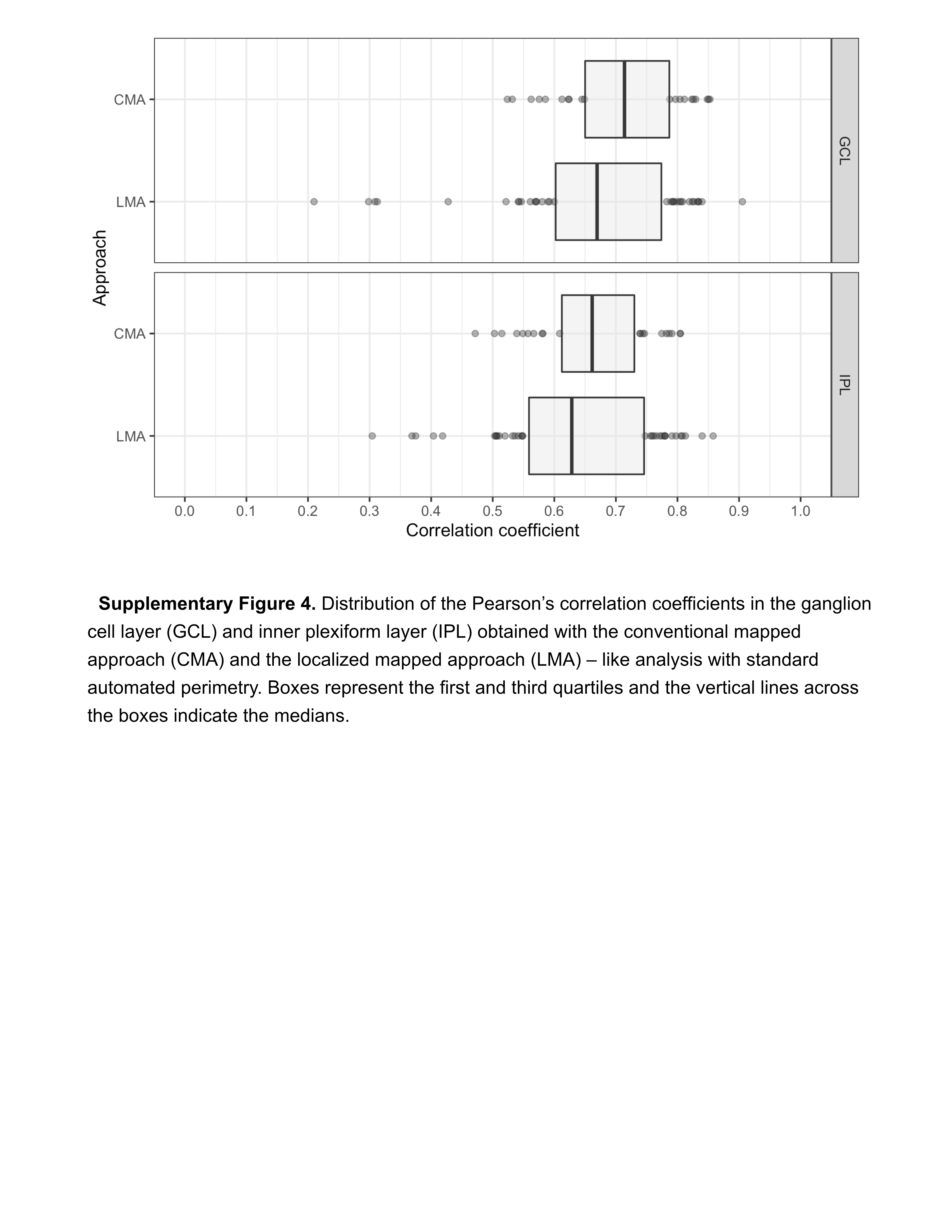

Supplement: Supplementary file 4 — Supplementary Information 4. [file 41598_2022_13730_MOESM4_ESM.tiff]

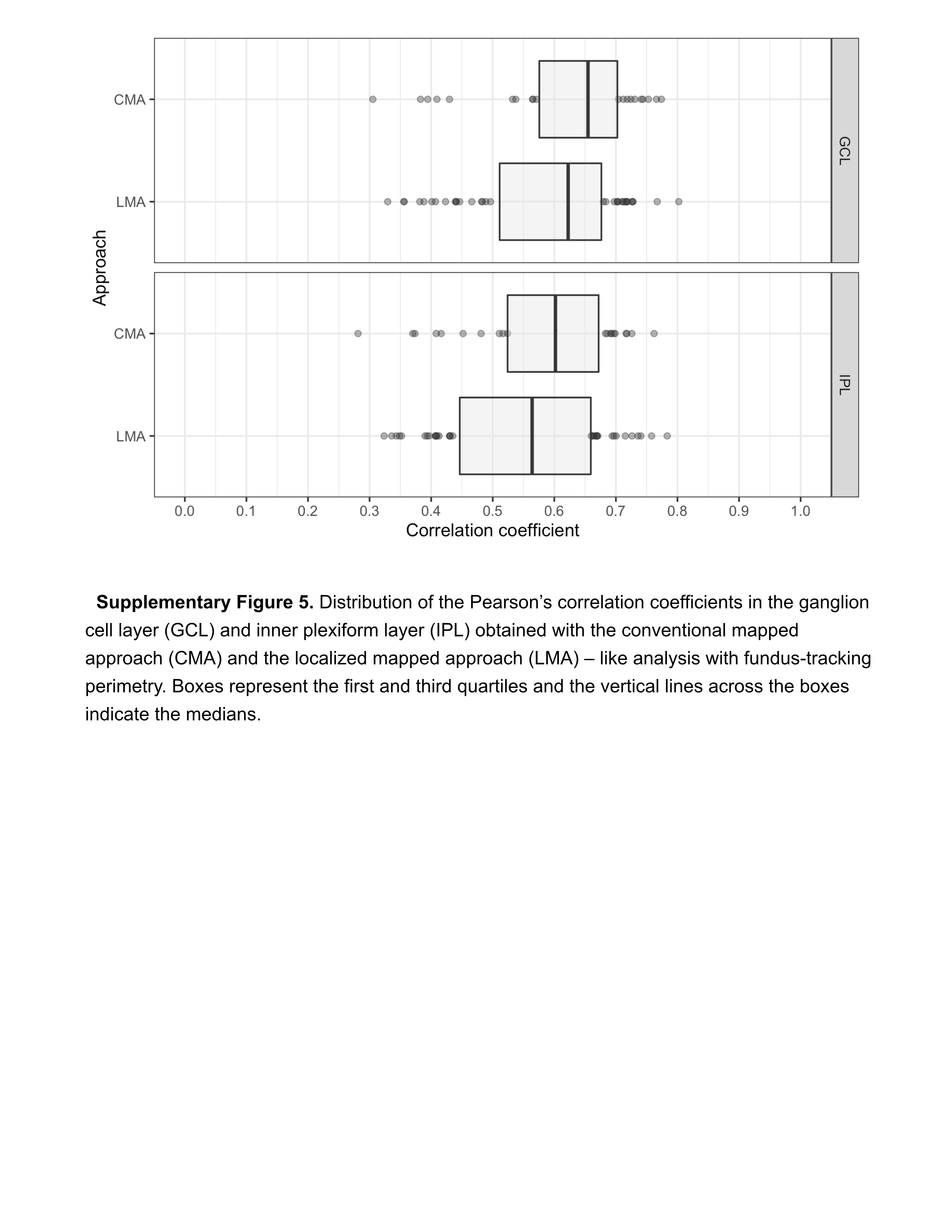

Supplement: Supplementary file 5 — Supplementary Information 5. [file 41598_2022_13730_MOESM5_ESM.tiff]

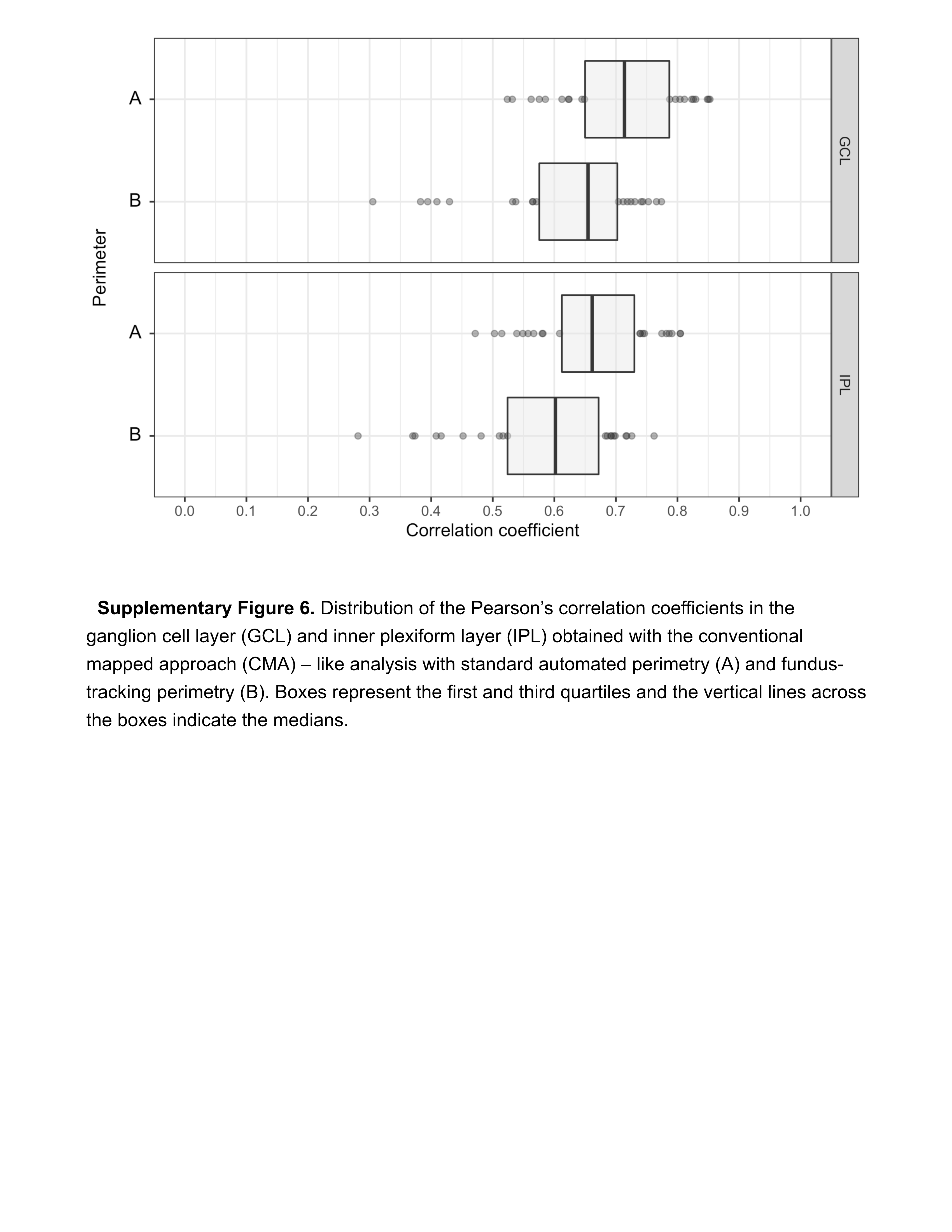

Supplement: Supplementary file 6 — Supplementary Information 6. [file 41598_2022_13730_MOESM6_ESM.tiff]

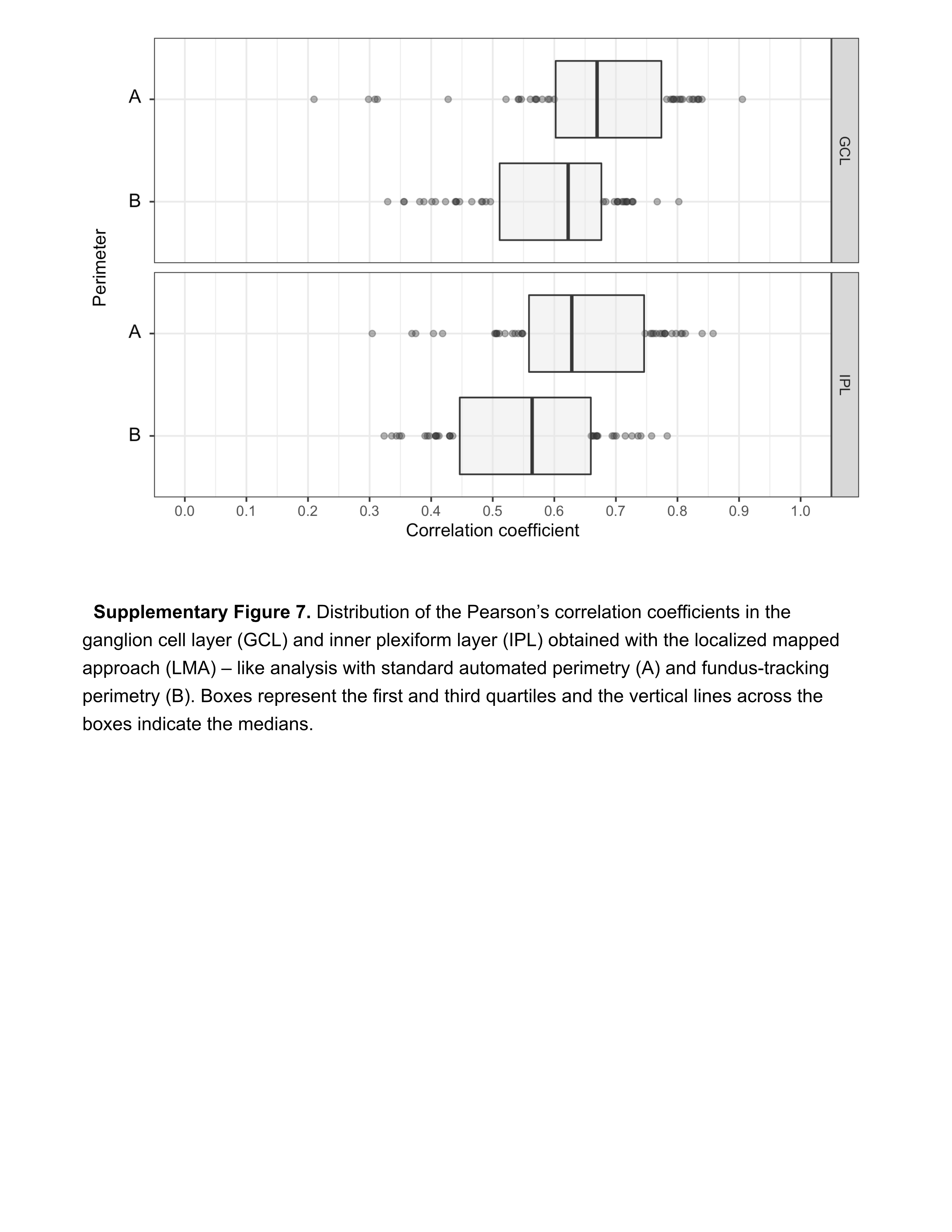

Supplement: Supplementary file 7 — Supplementary Information 7. [file 41598_2022_13730_MOESM7_ESM.tiff]

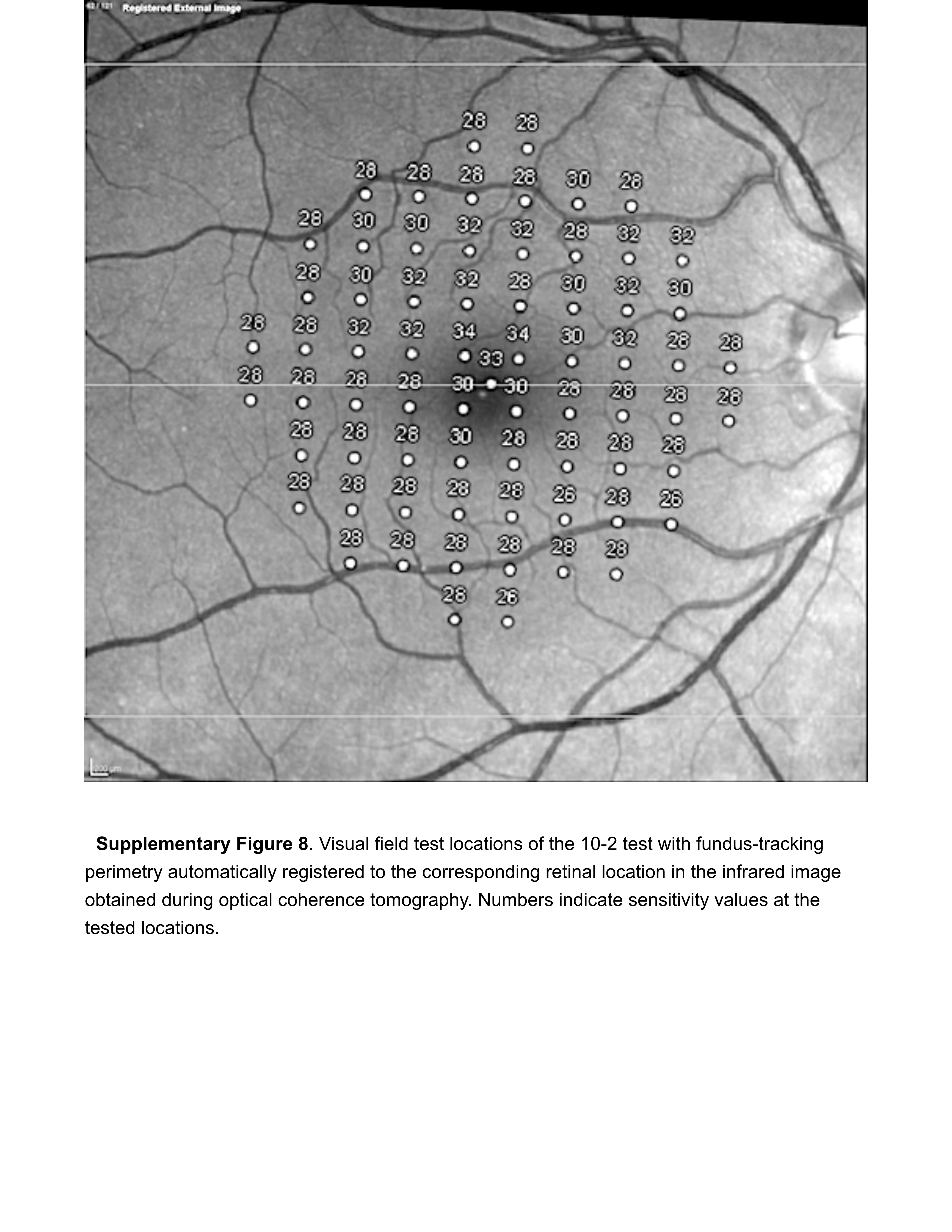

Supplement: Supplementary file 8 — Supplementary Information 8. [file 41598_2022_13730_MOESM8_ESM.tiff]
